# Recent Spatiotemporal Patterns of US Lung Cancer by Histologic Type

**DOI:** 10.3389/fpubh.2017.00082

**Published:** 2017-05-19

**Authors:** Denise Riedel Lewis, Linda W. Pickle, Li Zhu

**Affiliations:** ^1^Surveillance Research Program, Division of Cancer Control and Population Sciences, National Cancer Institute, Bethesda, MD, USA; ^2^StatNet Consulting, Gaithersburg, MD, USA

**Keywords:** lung cancer incidence, histology, trends, gender, race, geospatial modeling, micromap plots

## Abstract

**Background:**

After a period of increasing rates, lung cancer incidence is declining in the US for men and women. We investigated lung cancer rate patterns by gender, geographic location, and histologic subtype, and for total lung cancer (TLC), for the entire study period, and for 2000–2011 from 17 surveillance, epidemiology, and end results areas.

**Methods:**

For each gender–histologic type combination, time trend plots and maps of age-adjusted rates are presented. Time trend significance was tested by joinpoint regression analysis. Spatial random effects models were applied to examine effects of sociodemographic factors, health insurance coverage, smoking, and physician density at the county level. Linked micromap plots illustrate patterns for important model predictors.

**Results:**

Declining incidence trends occurred for TLC (*p* < 0.05, entire period). Squamous cell carcinoma trends increased for females only (*p* < 0.05). Small cell carcinoma trends declined overall, *p* < 0.05, but recently increased faster for females than males. Adenocarcinoma rates initially declined, but were significantly increasing by 2004, *p* < 0.05. Counties with higher current smoking and family poverty were strongly associated with higher risk for all gender–histologic types (*p* < 0.0001, for both variables). County socioeconomic status was associated with higher risk for all lung cancer subtypes for females, *p* < 0.02. Counties with more diagnostic radiologists were associated with higher TLC rates (*p* < 0.03); counties with greater primary care physician access were associated with lower TLC rates (*p* < 0.03). TLC incidence rates were higher in eastern and southern states than western areas. Male rates were higher than female rates along the West Coast. Males and females had similar small cell rate patterns, with higher rates in the Midwest and southeast. Squamous cell carcinoma and adenocarcinoma rate patterns were similar to TLC patterns, except for relatively higher female adenocarcinoma rates in the northeast and northwest.

**Conclusion:**

Geographic patterns and declining time trends for incident lung cancer are consistent with previous mortality patterns. Male–female time trend and geographic pattern differences occur by histologic type. Time trends remain significant, even after adjustment for significant covariates. Knowledge of the variation of lung cancer incidence by region and histologic type is useful for surveillance and for implementing lung cancer control efforts.

## Introduction

Lung cancer incidence and trends by histologic type in the US were recently described (1) for white and black populations from 1977 to 2010 and for white non-Hispanics, Asian/Pacific Islanders, and white Hispanics from 1992 to 2010. Lung cancer rates have been declining for both US men and women, with an overall decrease among men that became apparent in the early 1990s and among women since 2009. This paper extends the previous analyses by investigating whether the gender- and histology-specific rates differ across the Surveillance, Epidemiology, and End Results (SEER) registries, which represent 28% of the US population (2). To date, there have been few analyses of the geographic distribution of incident lung cancer in the US (3).

Smoking, the most important risk factor for lung cancer, was not studied in detail in the previous trend analysis. Analysis of data from the National Health Interview Survey (CDC 2015) (4) revealed that age-adjusted current smoking prevalence in adults 18 and older has fallen from 24.6% in 1997 to 15.1% in 2015 (January through June) in the US. Smoking prevalence is slightly higher in the age group 45–64 years for men and women (16.7%). Smoking is associated with poverty, education, race,^1^ and insurance status (5). Small cell carcinoma and squamous cell carcinoma are most associated with smoking; more modestly so for adenocarcinoma (6). Adenocarcinoma of the lung has been observed among non-smokers, leading to speculation that other potential environmental exposures may be important in the etiology of this subtype. Adenocarcinoma has been more predominant in women based on case series (7, 8). This paper presents an analysis of lung cancer incidence rate time trends and geographic patterns by gender and histologic type, including application of a regression model that includes important cofactors that might explain these patterns. Of particular interest is whether temporal and spatial patterns of lung cancer rates by histology differ between men and women, as the previous paper revealed some gender differences.

## Materials and Methods

**Figure 1:**
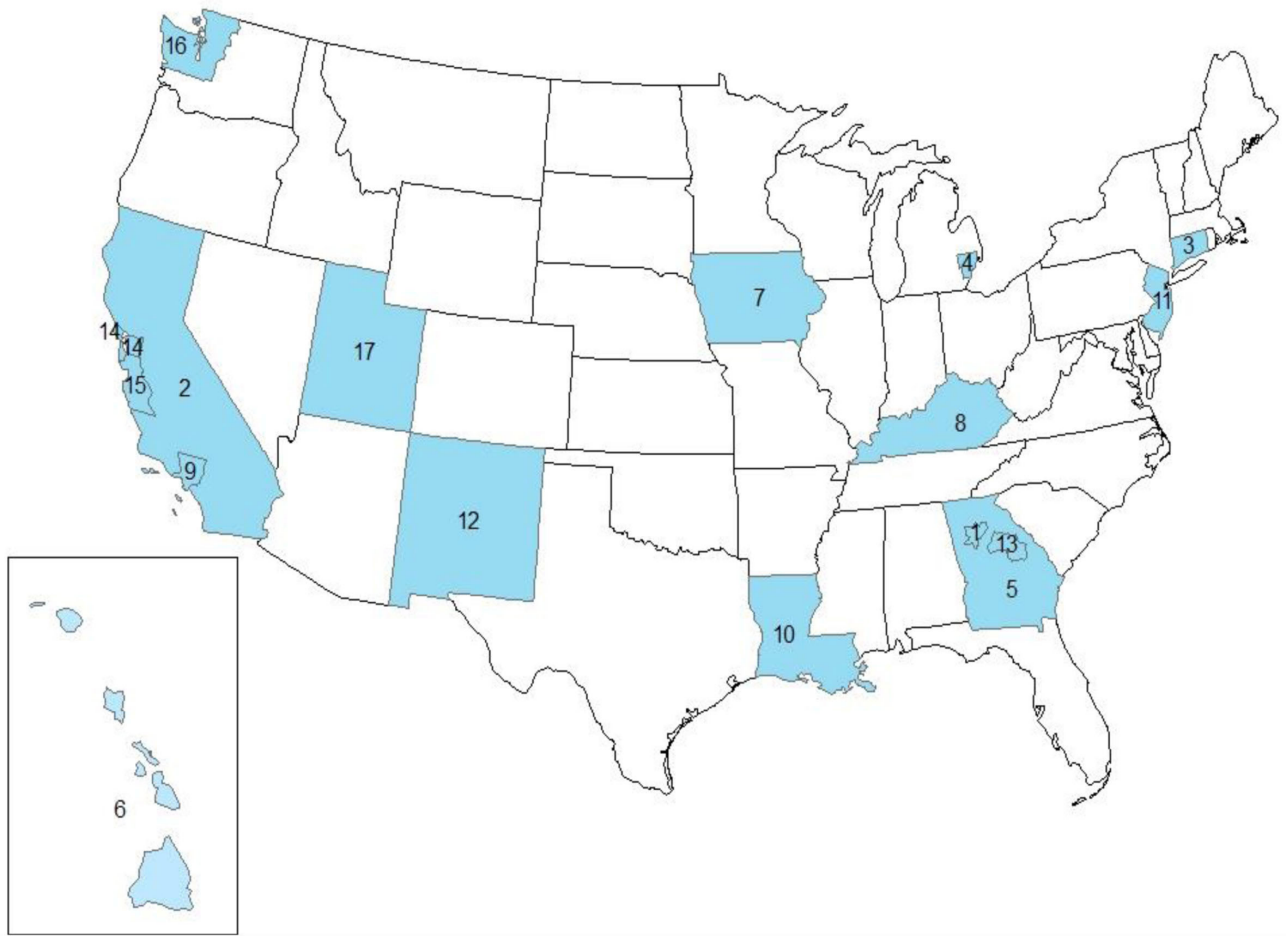
**Study area showing the SEER17 registries, excluding Alaska**. 1. GA-ATL, Atlanta (Metropolitan). 2. CA-OTH, California excluding CA-SF and CA-SJ. 3. CT, Connecticut. 4. MI-DET, Detroit (Metropolitan). 5. GA-OTH, greater Georgia. 6. HI, Hawaii. 7. IA, Iowa. 8. KY, Kentucky. 9. CA-LA, Los Angeles. 10. LA, Louisiana. 11. NJ, New Jersey. 12. NM, New Mexico. 13. GA-RUR, rural Georgia. 14. CA-SF, San Francisco-Oakland SMSA. 15. CA-SJ, San Jose-Monterey. 16. WA-SEA, Seattle (Puget Sound). 17. UT, Utah.

Data from the SEER program (2) were used in the analysis for 18 SEER registries (see Figure 1), excluding Alaska Natives, for the diagnosis years 2000–2011. Alaska Natives were excluded because this registry program does not include non-Native populations, i.e., non-Hispanic whites, non-Hispanic blacks, and other groups in the state of Alaska. Henceforth, we refer to these areas collectively as SEER17. SEER17 lung cancer incidence rates by gender and histologic type [total lung cancer (TLC), squamous cell carcinoma, small cell carcinoma, and adenocarcinoma] as established previously (1, 9) were examined individually and compared with incidence rates for the total SEER17 areas combined.

Incidence rates were age-adjusted by the Census 2000 US population for a total of 612 counties in the study area, covering approximately 28% of the US population. The 12-year study period was divided into two periods, 2000–2005 and 2006–2011, in the random effect regression models. Grouping into time periods allowed assessment of general time trends without assuming a linear trend for the rates, which would be implied by including single years of diagnosis in the model. SEER data include individual level data on the case type and other factors about the type of cancer (e.g., histology and stage), age at diagnosis, gender, race/ethnicity, and cancer registry. No other demographic or smoking data are collected for individual cases. Therefore, county specific variables (percent current smoking, percent of family below poverty, socioeconomic status in quintiles, and percent with no insurance, presence of diagnostic radiologists or physicians) are used to capture the characteristics of local environmental factors. While the county-level variables characterize the local environment on a population level, they do not necessarily reflect individual or case exposures to these factors.

The analysis is divided into several parts. First, time trend plots and maps of age-adjusted rates provide a visual sense of the rates. Time trends for each SEER area/gender/histologic type combination were tested for significant differences from the combined SEER area trends by joinpoint regression analysis (10). Finally, random effect models using SAS Proc Glimmix procedure (11) were run that included 11 main effects, two-way interactions, and spatial random effects for county-level variables. Random effects model fit was assessed by Akaike information criterion (AIC) and pseudo *R*^2^ statistics. Variograms of model residuals confirmed that all spatial autocorrelation was accounted for by the models. Linked micromap plots (12) illustrate the patterns for important county-level model predictors by histologic type and gender. These plots link graphs of rates and covariates to a series of small maps by color. The rows of the plot layout, representing the SEER17 areas, can be sorted by any variable, allowing examination of geographic clusters and trends associated with increasing (decreasing) values of the sort variable.

We used R version 3.1.3^2^ to initially examine the association between lung cancer incidence and the independent county-level variables. Assumptions required of the proposed generalized linear mixed effect models were checked. The age-adjusted incidence rates were approximately normally distributed, so no transformation was necessary. Lung cancer incidence rates were weighted in the model by county population size to stabilize their variances for large and small counties. Prior to running the models, we did a correlation analysis to select the appropriate main effects for the model. Separate models were fit for males and females and for each of the four lung cancer histology types. Because we were primarily interested in the more defined non-small cell (primarily adenocarcinoma and squamous cell carcinoma), and small cell carcinomas, we did not model other specified carcinomas, or carcinomas with unspecified histologic type.

Main effect variables were chosen to see whether there were smoking, race/ethnicity, or other social determinants that might help explain the variance of the lung cancer incidence rates by histologic type. The 11 main effects included covariates for time period (2000–2005 and 2006–2011), and county specific variables for smoking status, socioeconomic status (13), poverty, access to health insurance, physician density, and county demographic characteristics (Table 1). The data sources for the covariates are listed in Table 1. The race-ethnic variables reflect the percent composition of the county population for the particular race-ethnic group and are based on a continuous scale of composition. Socioeconomic status is categorized into high, medium, and low SES according to a composite index based on census tract information. Important two-way interactions between the covariates were selected using the Elastic Net approach, a weighted-average of the least absolute shrinkage and selection operator (LASSO), and Ridge regression variable selection methods (14). Interactions from this initial variable selection process were retained in the subsequent models only if they were significant at the.05 level. Because of this restriction, final models included different interactions for the different gender and histologic type combinations.

**Table 1:** **Variables, Surveillance, Epidemiology and End Results (SEER) registry, and county-level data sources**.

Variables	Definition	Data sources
**Dependent variable**		
Lung cancer histologic types (4): total lung, squamous cell carcinoma, small cell carcinoma, and adenocarcinoma	Age-adjusted incidence rates of lung cancer histologic type separately for males and females ages 20 and older. Rates are incident cases per 100,000	SEER, http://www.seer.cancer.gov/data
**Predictor variables**		
Period	2000–2005 and 2006–2011	Periods are based on year of diagnosis with lung cancer histologic type in the SEER data
Percent of current smoking	Current smoking prevalence during 1997–1999 and 2000–2003 among people 18 years of age or older	Small area estimates, http://sae.cancer.gov
Percent of family below poverty level	Percent of families whose incomes are below the poverty level	Census 2010
SES composite index	County-level SES index constructed using the data from Census 2000 and ACS 2005–2009 (15)	Yu et al. (13)
Density of diagnostic radiologists	Number of diagnostic radiology specialists in 2010 per 1,000 population normalized by the total population in 2010	HRSA Area Resource File (ARF), http://arf.hrsa.gov/, accessed *via* SEER*Stat (16)
Density of primary care physicians	Number of primary care physicians in 2010, per 1,000 population, normalized by the total population in 2010	HRSA ARF, http://arf.hrsa.gov/, accessed *via* SEER*Stat (16)
% Black	Percent of total population that is black in 2010	US Census Bureau 2010, http://www.census.gov/2010census/
% American Indians	Percent of total population that is American Indian in 2010	US Census Bureau 2010, http://www.census.gov/2010census/
% Asian Pacific Islander (API)	Percent of total population that is API in 2010	US Census Bureau 2010, http://www.census.gov/2010census/
% Hispanic	Percent of total population that is Hispanic ethnicity in 2010	US Census Bureau 2010, http://www.census.gov/2010census/
% Uninsured	Percent of total population, ages 40+ with no health insurance coverage in 2010	US Census Bureau 2010, http://www.census.gov/2010census/

We started with fixed effect models that accounted for the main effects and the selected two-way interactions. The standardized residuals from the fixed effect models showed a strong spatial distribution pattern in variogram plots (17) for all the gender and histologic type combinations, suggesting a random effect model accounting for the spatial autocorrelation in the lung cancer incidence rates. We then modified the model to include a spatial random effect with an exponential covariance structure, based on the variogram plot of the standardized residuals. We considered what statistical tests were available to adjust for multiple comparisons including assessing the false discovery rates. However, none of these have been shown to be appropriate for spatially autocorrelated data as the resulting *p* values from our analysis are not independent. Therefore, we did not adjust for multiple comparisons. We assessed model fit using AIC, pseudo *R*^2^, and residual analysis. AIC is a measure of the log-likelihood with a penalty for every added covariate (18), with a smaller value suggesting better fit. Pseudo *R*^2^ measures the proportion of total variance in the rates explained by the model, and a large value suggests a better fit. To compare the fixed effect model and the random effect model, a better fit is in the model with the higher pseudo *R*^2^ and the lower AIC value.

## Results

The incidence rates among males decline over time for most subtypes, except for a slight rise in adenocarcinoma beginning in 2004. The female incidence rates by subtype remain steady or decline slightly, except for adenocarcinoma which has a long steady increase beginning in 2005, with a marked rise at the end.

**Figure 2:**
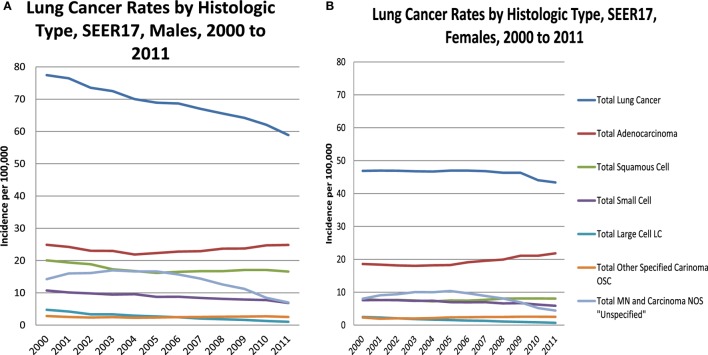
**Lung cancer rates by histologic type, males (A) and females (B), 2000–2011, SEER17, excluding Alaska**.

In Figures 2A,B, lung cancer incidence rates by histologic type are presented for males and females by year of diagnosis from 2000 to 2011. For TLC, male rates decline steadily from 2000 forward, consistent in a subset of SEER areas (1). Female rates are steady from 2000 to 2009, when they begin a modest decline. Both males and females have a decline in the total malignant neoplasm and carcinoma not otherwise specified (NOS), also known as the “unspecified” group, in the mid 2000s as this was a time when immunostaining for TTF-1 was introduced by pathologists (1). Other immunohistochemical markers were introduced for squamous cell carcinoma differentiation, including p63 and p40 (19–22), which also may explain the slight increase among females and the moderating decline among males.

Table 2 shows the incidence rates and trends [annual percent change (APC)] for lung cancer by histologic type and gender. Results are shown by temporal or year groupings where joinpoint regression identified significant changes in time trends. TLC has been declining for males and females, especially since 2009. Male and female trends differ by histologic site with large declines in the earlier period for males for the three histologic subtypes, squamous cell carcinoma, small cell carcinoma, and adenocarcinoma. Among males, there were slight increases for squamous cell starting in 2005 (non-significant) and adenocarcinoma starting in 2004 (significant increase). Female rates for TLC and the subtypes squamous cell carcinoma, small cell carcinoma, and adenocarcinoma had modest declines compared to the male rates. Unlike males, female squamous cell carcinoma began to rise in 2004 (significant), while small cell rates had a steep decline beginning in 2009 (significant). Male and female adenocarcinoma rates began increasing in 2004, although the rate of increase was greater for females (APC = 2.8%, significant), as compared with males (APC = 1.8%, significant).

**Table 2:** **Incidence trends for lung cancer histologic subtypes, Surveillance, Epidemiology, and End Results 17, excluding Alaska, by gender, 2000–2011**.

Lung histologic subtype	Male				Female			
	Rate	APC	APC 95% confidence interval	Period	Rate	APC	APC 95% confidence interval	Period
Total lung cancer	70.1	−2.0*	(−2.3 to −1.8)	2000–2009	46.7	−0.2*	(−0.3 to 0.0)	2000–2009
	61.7	−4.0*	(−6.9 to −1.0)	2009–2011	44.6	−3.7*	(−5.0 to −2.2)	2009–2011
Squamous cell carcinoma	18.1	−4.1*	(−5.3 to −2.9)	2000–2005	7.6	−1.8	(−3.6 to 0.0)	2000–2004
	16.7	0.7	(−0.2 to 1.6)	2005–2011	7.8	1.7*	(0.9 to 2.4)	2004−2011
Small cell carcinoma	8.8	−3.4*	(−3.9 to −2.9)	2000–2011	7.1	−1.7*	(−2.2 to −1.1)	2000–2009
					6.3	−6.1*	(−11.6 to −0.1)	2009–2011
Adenocarcinoma	23.4	−3.1*	(−4.3 to −2.0)	2000–2004	18.3	−0.7	(−0.7 to −1.9)	2000–2004
	23.4	1.8*	(1.3 to 2.3)	2004–2011	19.9	2.8*	(2.4 to 3.3)	2004–2011
Large cell	2.5	−11.8*	(−12.8 to −10.7)	2000–2011	1.8	−9.1*	(−9.9 to −8.3)	2000–2007
					1.0	−13.1*	(−15.6 to −10.5)	2007–2011
Other specified carcinomas	2.6	−9.0	(−20.7 to −4.3)	2000–2002	2.3	2.3*	(1.3 to 3.2)	2000–2011
	2.5	1.6*	(0.4 to 2.9)	2002–2011				
Total malignant neoplasms and carcinoma unspecified	16.1	2.9	(−0.4 to 6.2)	2000–2005	9.5	4.6*	(1.2 to 8.2)	2000–2005
	14.0	−10.1*	(−16.5 to −3.2)	2005–2009	9.2	−8.2	(−20.7 to 6.2)	2005–2008
	8.9	−22.2*	(−35.7 to −5.9)	2009–2011	6.1	−18.9*	(−26.1 to −11.0)	2008–2011

*APC, annual percent change*.

*Change is significantly different from 0, p < 0.05.

**Figure 3:**
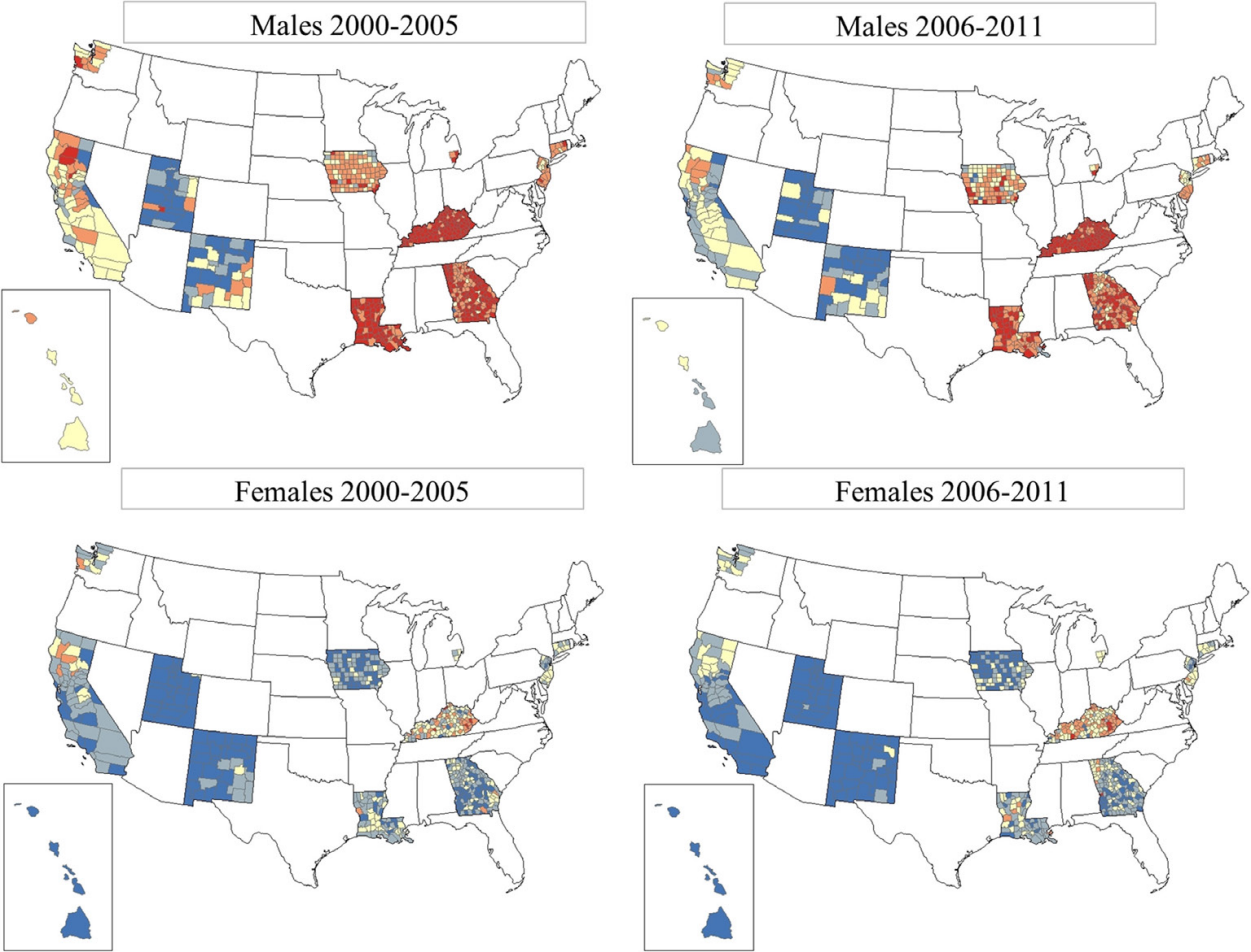
**Total lung cancer incidence rates by county for SEER17, excluding Alaska**. From top row left: total lung cancer for males, period 1 (2000–2005); top row right: total lung cancer for males, period 2 (2006–2011). Bottom row left: total lung cancer for females, period 1 (2000–2005); bottom row right: total lung cancer for females, period 2 (2006–2011). **SEER17_counties rate** 
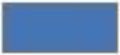
 **0.00–41.82,** 
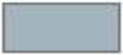
 **41.83–53.53,** 
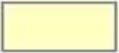
 **53.54–68.15,** 
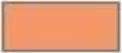
 **68.16–91.96, and** 
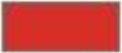
 **91.97–199.10**.

Figure 3 shows the TLC rates by county for gender and period, 2000–2005 and 2006–2011. It is clear that males have higher TLC rates that are more pronounced in the southern SEER areas, for example, in Kentucky, Louisiana, and Georgia. TLC rates appeared to decline in all SEER areas in 2006–2011, as evidenced in the western areas and eastern US. Rates improved in the south, but continued to be among the highest in the more recent period. Among females, rates for TLC were low and declined in the more recent period. However, in certain counties of Kentucky, Louisiana, and Georgia, rates of TLC for females increased. Geographic patterns and time trends for incident lung cancer are consistent with mortality patterns, see Figure S1 in Supplementary Material showing TLC mortality rates for gender and period for the US.

Table 3 shows the random effects regression model results for 11 main effects for county characteristics for TLC, squamous cell carcinoma, small cell carcinoma, and adenocarcinoma by gender. The model results showed better fit with the inclusion of two socioeconomic variables (family poverty and percent no insurance) for males and three socioeconomic variables (family poverty, percent no insurance, and socioeconomic status) for females, with lower AIC values. Negative coefficients for period indicate incidence rates are declining for TLC incidence (male and female), male squamous cell carcinoma, and male and female small cell carcinoma incidence. Incidence rates appear to be increasing for female squamous cell carcinoma, and male and female adenocarcinoma. For current smoking, the regression coefficients across all the histologic types are positive, indicating a strong positive association with current smoking (*p* < 0.01). Family poverty coefficients are consistently positive, indicating that lung cancer incidence across all histologic types is significantly associated (*p* < 0.01) with a greater level of poverty for both males and females. Coefficients for percent no insurance are mostly negative, indicating that areas with more people who were not covered by health insurance had lower lung cancer rates. Significance for percent insurance ranged from *p* < 0.0001 (male squamous cell, and male and female adenocarcinoma) to *p* = 0.04 (TLC), where it was significant at *p* < 0.05, suggesting that lack of insurance is more of a risk factor for some subtypes of lung cancer than others. Higher socioeconomic status (shown in the random effects models for females only) is associated with higher rates, especially for TLC, and adenocarcinoma. Squamous cell carcinoma and small cell carcinoma are also associated with high SES, but not to the same magnitude as the other types. Diagnostic radiologist county density coefficients are only slightly positive across all the subtype and gender models (significant, range *p* < 0.0001 to *p* < 0.03), indicating more diagnostic radiologists in areas with a higher lung cancer incidence. Physician county density coefficients are negative across all of the lung cancer subtypes, indicating that areas with more physicians have a lower lung cancer incidence (significant, range *p* < 0.0001 to *p* < 0.03). Counties with a higher percent of Hispanics generally have lower incidence rates. The exception to this is the higher adenocarcinoma rate for male and female Hispanics. Counties with a higher proportion of black residents have significantly declining rates for all types and both genders, except for adenocarcinoma. Adenocarcinoma rates for black males increased slightly while rates for black females were steady. Lung cancer incidence rates declined for all histologic types in counties with higher proportions of American Indian/Alaska Natives. Rates in counties with a sizeable Asian Pacific Islander (API) population declined for all types except adenocarcinoma, which had steady rates for males and a slight increase for females. Rates in counties with a sizeable Hispanic population also declined, except for adenocarcinoma which had slight increases for both males and females.

**Table 3:** **Results of random effects regression models for incident lung cancer histologic type by gender**.

Effect	Total lung cancer				Squamous cell				Small cell				Adenocarcinoma			
	Male		Female		Male		Female		Male		Female		Male		Female	
	Coeff	Sig	Coeff	Sig	Coeff	Sig	Coeff	Sig	Coeff	Sig	Coeff	Sig	Coeff	Sig	Coeff	Sig
Period	−7.86	**	−4.64	**	−1.07	**	0.70	**	−2.31	**	−0.48	**	0.93	**	2.76	**
Current smoking	1.45	**	0.52	**	0.67	**	0.36	**	0.40	**	0.35	**	0.46	**	0.49	**
Family poverty	0.96	**	0.74	**	0.62	**	0.32	**	0.23	**	0.23	**	0.30	**	0.38	**
SES			4.45	**			0.95	**			0.42	*			3.02	**
Percent (%) no insurance	−0.40	*	0.03	NS	−0.38	**	−0.10	*	−0.23	**	−0.06	NS	−0.77	**	−0.44	**
Diagnostic radiologist	0.05	**	0.06	**	0.02	**	0.02	**	0.01	*	0.02	**	0.02	**	0.03	**
Physician	−0.26	**	−0.22	**	−0.12	**	−0.07	**	−0.04	**	−0.07	**	−0.05	*	−0.05	**
Black	−0.14	**	−0.11	**	−0.03	*	−0.02	**	−0.07	**	−0.05	**	0.05	**	0.00	NS
American Indian/Alaska Native	−1.00	**	−0.51	**	−0.27	**	−0.16	**	−0.18	**	−0.14	**	−0.16	**	−0.12	**
Asian Pacific Islander (API)	−0.52	**	−0.42	**	0.02	NS	−0.03	**	−0.04	**	−0.03	**	0.00	NS	0.03	*
Hispanic	−0.40	**	−0.25	**	−0.10	**	−0.04	**	−0.07	**	−0.05	**	0.04	*	0.04	*
**Interactions**																
American Indian × API	0.52	**	0.23	**	−0.18	**										
Period × current smoking			0.24	**												
Period × % no insurance									0.05	*						
Pseudo *R*^2^	0.61		0.53		0.37		0.24		0.30		0.31		0.22		0.16	

*Results shown without the intercept*.

Coeff, coefficient; Sig, statistical significance; **p < 0.01; *p < 0.05; NS, non-significant.

*Model results for males include two socioeconomic (SES) variables: family poverty and percent no insurance at the county level*.

*Model results for females include three socioeconomic (SES) variables: family poverty, socioeconomic status, and percent no insurance at the county level*.

Overall, the magnitudes of the main effects coefficients were similar for TLC, squamous cell carcinoma, and small cell carcinoma for males. However, male adenocarcinoma results were slightly different in that the period coefficient was positive. The coefficients for black race and Hispanic ethnicity were positive, indicating an increase in adenocarcinoma rates over time, and higher rates for blacks and Hispanics, compared to non-Hispanic whites. The magnitude of the coefficients of the lung cancer histologic models for females was similar for squamous cell carcinoma and small cell carcinoma except for a difference in time trend. Negative period coefficients indicate a decline in total rates and small cell rates over time. Squamous cell carcinoma rates had a slight increase and adenocarcinoma rates had a large increase among females. The trend analysis in Table 2 indicates that both of these increases began around 2004. The SES coefficient was significantly positive in all of the female models indicating higher rates in areas with higher SES levels. This effect was particularly strong for both TLC and adenocarcinoma.

**Figure 4:**
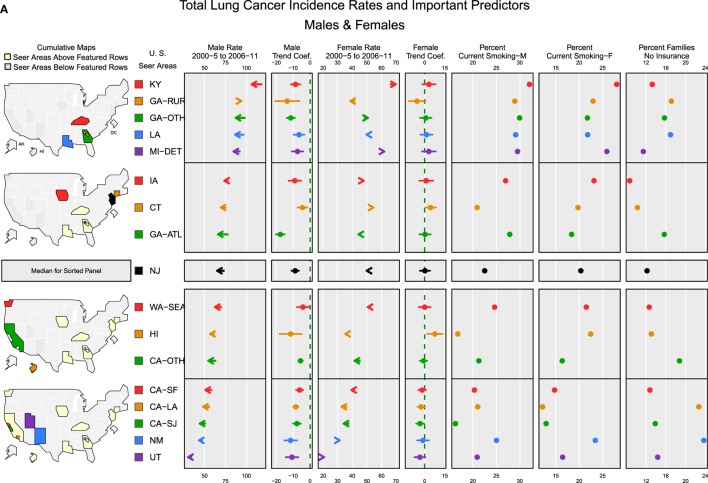

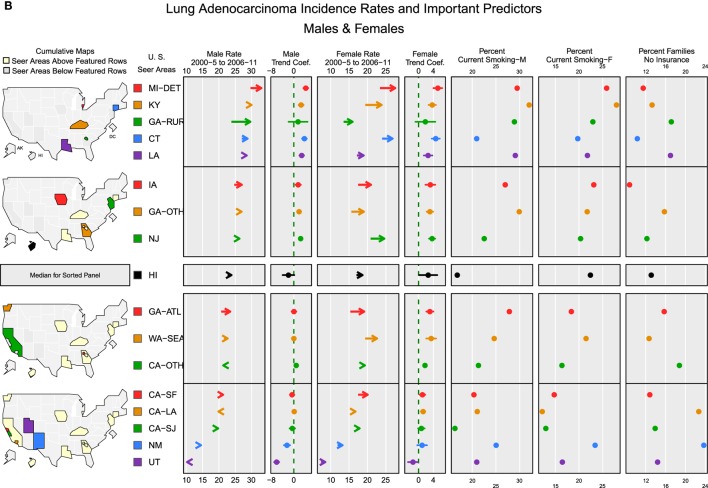
**Incidence rates and important predictors for (A) total lung cancer and (B) adenocarcinoma**. **(A)** The covariate values shown are population-weighted averages across counties in each registry. Arrows indicate the direction of change in rates from 2000–2005 (tail) to 2006–2011 (arrowhead). **(B)** The covariate values shown are population-weighted averages across counties in each registry. Arrows indicate the direction of change in rates from 2000–2005 (tail) to 2006–2011 (arrowhead).

Figures 4A,B micromap plots (12) illustrate how some of the important predictors in the random effects models for TLC and adenocarcinoma (23) are associated with the incidence rate of lung cancer in each SEER region. Rates are displayed with the trend coefficients from the random effects model along with 95% confidence intervals testing whether the trend is significantly different from 0. The registry-specific incidence rates for TLC and adenocarcinoma are sorted by the order of the male rates for the later period, 2006 to 2011. The plots display the population-weighted means for smoking and no insurance, several of the most important model covariates across all counties in each registry area. In general, both plots show higher rates in eastern and Midwestern SEER registries and lower rates in the western SEER registries. Registry-specific TLC rates generally follow the same order from highest to lowest for males and females. For TLC (Figure 4A), all of the male rates across the registries are declining significantly, after adjusting for important covariates in the model. For females, most registries have small, non-significant trends. The exceptions are San Jose, which has a significant decline, and Connecticut and Hawaii, which had significant increases. Based on the direction of the trend arrows in the figure, rates and trends for TLC are diverging in Kentucky, greater Georgia, Detroit, Iowa, Connecticut, New Mexico, and Utah. That is, female rates are increasing and male rates are decreasing in these areas, although the increase among females is only significant in Connecticut and Hawaii. Smoking rates are generally higher in high rate areas. Female smoking rates appear to be negatively correlated with the percent of families with no health insurance, i.e., smoking rates are higher in areas with high insurance coverage, with the exception of New Mexico that has a high percentage of no insurance coverage and a higher percent of current smoking.

For adenocarcinoma (Figure 4B), the rank order for male and female rates by registry also follows a similar pattern with a few exceptions. Most of the SEER areas with the higher rates also have higher percentages of current smoking. Overall, areas with high adenocarcinoma rates for both males and females have significantly increasing rates, possibly reflecting the increased use of diagnostic markers and improvements in histologic subtyping (1). Areas with adenocarcinoma rates below the median rate for males have trends that are no different from 0, except for a slight increase in greater California and significant decreases in New Mexico and Utah. For females, most areas have significantly increasing rates except for Utah, where rates are declining significantly. In all areas where both male and female rates have increasing trends, the female trend is stronger (i.e., a larger trend coefficient), except in Louisiana where the trends are nearly equal. In San Francisco, San Jose, Hawaii, and New Mexico, adenocarcinoma rates are converging by gender, with higher male rates decreasing and lower female rates increasing. The plots for percent families with no insurance show that the places where percent of residents lacking insurance coverage is among the lowest (Detroit, Kentucky, Connecticut, New Jersey, and Iowa), adenocarcinoma rates for both males and females are above the median rate and are increasing.

## Discussion

This new analysis of lung cancer incidence rates by histologic type expands upon a previous analysis (1) by using data from SEER17 (SEER18 excluding Alaska). The previous analysis did not include the SEER expansion registries of the rest of California outside of San Francisco and San Jose-Monterrey, greater Georgia, Kentucky, Louisiana, and New Jersey. This analysis increases coverage of the US population by approximately 18%. The 2014 analysis relied on a new pathologic classification of lung cancer (21) that was first implemented by the SEER program in 2013 (24). Our new analysis including geospatial factors, based on the revised histologic classification, used a fourfold approach: first, using visual inspection of time trends (using graphs) and geographic patterns of rates by histologic type and county (using maps); a joinpoint analysis of time trends by histologic type versus total SEER17 trends; random effects regression models with the rate of the histologic type as the dependent variable and covariates at the county level; and finally linked micromap plots and trend coefficients to illustrate patterns for important model predictors based on the random effects model results.

A major strength of the current analysis is the high quality of SEER incidence data. High quality data parameters include large coverage of the US population, frequent data quality checks of the SEER data, and high measures of case completeness for cancer sites in the SEER data (equal to or more than 98% over a 10-year period). SEER data are not a statistically random sample of the US population; however, SEER data do cover 28% of the US population and are more varied by race/ethnicity and SES than the collection of SEER9 data. All cases in this study were microconfirmed, cases diagnosed by death certificate only were excluded, and all had histologic typing performed. Improved histologic typing for lung cancer was recently implemented by the SEER program in light of the updated World Health Organization classification of tumors of the lung and other respiratory sites (25). SEER data are reliant on individual pathologists for histologic coding; however, certified tumor registrars provide additional review for coding histologies. SEER registry data are also subject to data quality reviews, with fewer than 2.5% of non-specific histologic codes permitted at the time of data submission to the SEER program. The increased specificity of lung cancer histologic type is the result of these data quality measures. The high quality of the data plus greater coverage of the US population suggests that the analytic findings most likely represent the lung cancer patterns over the entire US population.

Inclusion of more SEER registry areas suggests that the increase in adenocarcinoma may be present in a larger proportion of the population as compared with the population in the analysis in Lewis et al. (1). In addition, all the SEER registry sites showed an increase in trend for adenocarcinoma among females, except for Utah, which could reflect the slower decline in smoking among females (26, 27), and possibly continuing exposure to second hand smoke (SHS) (28). Given that each histologic type of lung cancer was associated with higher rates of current smoking in the random effects models, there is the potential for an association with exposure to more SHS in these geographic areas. The composition of cigarettes has changed, resulting in deeper inhalation that may have contributed to the decrease in squamous and small cell carcinomas of the central airways and increased the peripheral adenocarcinomas associated with deeper inhalation (1). At the same time, trends for histologic types that are less well described, particularly large cell carcinoma and total malignant neoplasms and carcinoma NOS (“unspecified”), continued to fall, as more lung cancer tumor types have been coded to more definitive types in recent years (1).

Another new aspect of our analysis was the addition of random effects models that account for spatial correlation among counties in a registry area. Most previous lung cancer analyses have ignored spatial correlation, but this can lead to misspecified significance levels and thus to incorrect inferences about the data. The model results add more evidence that adenocarcinoma rates have different associations with local factors compared to other lung cancer subtypes. A primary finding, among females, is an association of higher rates with a higher level of SES, perhaps pointing to a non-tobacco exposure, or perhaps some other environmental exposure or behavioral characteristic. Despite high SES in some areas, like Connecticut, TLC and adenocarcinoma rates are rising. Connecticut has significantly less primary care physicians, with a high number of diagnostic radiologists. Perhaps even with good primary care, detection of (adenocarcinoma) lung cancer is missed.

Our random effects models found that smoking was positively associated with each of the lung histologies, consistent with a previous study (6). Risk declines with smoking cessation more rapidly for squamous cell carcinoma and less rapidly for adenocarcinoma (28). Most newly diagnosed lung cancer cases have been among former smokers recently (29), which could influence the histologic trends and the type of lung cancer at first diagnosis. Access to physicians was associated with lower rates of all types of lung cancer histologies and access to diagnostic radiologists is important for lung cancer screening and detection. The strong positive association found between higher rates and current smoking prevalence is consistent with the continued importance of smoking, and the potential for individual exposure to SHS.

The micromap plot display for the TLC and adenocarcinoma type confirms previous associations of higher lung cancer rates associated with smoking and poverty. However, there is a striking difference for adenocarcinoma among females, in that lower SES is associated with high adenocarcinoma rates in some areas of the south and northeast, but high SES is associated with moderate rates of adenocarcinoma in the coastal west and in the northeast (results not shown). These positive trends are occurring regardless of current smoking status and other measures of SES.

A first limitation of the analysis is that cofactors were measured at an aggregate (county) level, single time, and not at a personal level. Examples include SES and percent current smoking based on small area estimates, not individual estimates. Urban and rural residence was not included due to the correlation with SES, where rural counties tend to reflect lower SES. The smoking period occurred immediately before the lung cancer diagnosis years, which did not allow for a longer period of exposure prior to lung cancer incidence. However, the strongly significant smoking coefficients from the random effects models for each lung cancer subtype support increased lung cancer rates by histologic type and the known relationship of smoking and lung cancer despite an imperfect smoking measure. Time trends are those that occur after all cofactors were entered into the random effects models. The cofactors contributing to the model are measured at one point in time. These trend effects are measured at the county level, not at the individual level.

Rates for histologic lung cancer types have been imputed in a recent analysis (30) to correct for non-specific histology. That paper uses data from SEER9, a slightly different histologic coding scheme and a multiple imputation method. Results of that analysis indicate that after imputation, adenocarcinoma rates decreased for men but showed a continuously increasing trend for women. However, both the Lewis et al. (1) and Yu et al. (30) analyses indicate changes in trends for lung cancer types that were NOS. Future studies could incorporate the Yu imputation and the histologic groupings used in Lewis et al. to examine the effect on more specific histologic coding on time trends for specific histologic types.

A second limitation of this study is that we were not able to consider air pollution or particulate matter exposure in the analysis. Most particulate matter studies have concentrated on non-lung cancer outcomes. A study by Dominici et al. (31) describes mapping particulate matter and evaluating geographic variation in mortality of total mortality, cardiovascular-respiratory mortality, and other causes of mortality. Data on particulate matter concentrations less than 10 µm in aerodynamic diameter (AD) (PM_10_) were used from 88 cities from the National Morbidity, Mortality, and Air Pollution Study from 1987 to 1994 (32, 33), a time prior to the lung cancer incidence rates we evaluated. Dominici et al. (31) note that particle composition differs depending on geographic region, with smaller particulates of less than 2.5 µm in AD (PM_2.5_) dominating in the eastern US with more sulfates and less organic carbon. Crystal dusts are more common in agricultural and desert areas. We note that some of the SEER areas in our study with an increase in lung cancer incidence among females in the upper Midwest and northeast were also areas of increased mortality from other causes in the Dominici paper. We noted decreasing TLC incidence in California; however, adenocarcinoma rates are low, but increasing, for females throughout California. Another study by Zeger et al. (34) evaluated all causes of mortality in a Medicare population with exposure to fine particulate matter (PM_2.5_) from 2000 to 2005 from the US EPA’s AirData database. Results of the US map of the PM_2.5_ data from this study showed a large distinct area of the eastern US with higher PM_2.5_ concentrations and the largest concentrations of PM_2.5_ found in the central valley region of California. Increased associations for all causes of mortality were noted mostly in the eastern and central regions of the US. A previous study by Pope et al. (35) using American Cancer Society Cancer Prevention Study II analyzed the mortality of 1.2 million adults prospectively across all 50 states from 1982 to 1988. Exposure estimates were assigned to each participant based on address at enrollment and the 3-digit zip code of residence using air monitoring data from various sources across the US. Fine particulate pollution was associated with a significant increase in lung cancer mortality for the exposure periods from 1979 to 1983 and 1999–2000 of 8 and 13%, respectively, after controlling for age, sex, race, smoking, education, marital status, body mass, alcohol consumption, occupational exposure, and diet. A recent study by Correia et al. found that declining concentration of particulate matter reduced premature mortality (36). Another recent study by Gharibvand et al. evaluated the association between ambient PM_2.5_ and lung cancer among never smokers (37). Most of the lung cancer case types (66.4%) were adenocarcinomas. The results indicated an increase in lung cancer risk for each 10-µg/m^3^ increase in ambient PM_2.5_ concentration. Risk estimates for lung cancer were higher for those with longer time at their enrollment address and if they spent more than 1 h per day outdoors.

While our analysis did not consider air pollution, the results from the particulate matter analyses support the plausibility for an increased risk in lung cancer histologic types not usually associated with tobacco products. Our results noting the difference in the direction of rates by histologic type, after controlling for demographic factors and smoking, may provide a further clue to the risk for adenocarcinoma in light of previous particulate matter research. Future studies that evaluate adenocarcinoma and other lung histologic subtype trends should consider specific airborne environmental exposures in addition to SES factors related to level of available health care and tobacco exposure and the more inclusive approach to coding lung cancer histologic type (1).

## Conclusion

We have applied a geospatial analysis and random effects modeling to explain differences in incidence rates among histologic types by gender, demographic, and socioeconomic factors. The geographic patterns for incident TLC rates were very similar to those of lung cancer mortality rates, as expected for a disease that is highly and rapidly fatal. A previous analysis of lung cancer mortality rates in the US using National Center for Health Statistics modeled data showed a cluster of high lung cancer mortality for white females on the west coast in the 1980s and 1990s, but only for the oldest age groups (38). This suggested a cohort effect of declining rates for younger age groups, perhaps due to reduced smoking rates in younger women. This high rate west coast cluster is no longer evident for either gender.

In all, this latest analysis confirms lung cancer histologic trends continuing to decline for males and females. However, there are a few SEER areas where there are increasing trends with higher rates for TLC for females in Kentucky, greater Georgia, Detroit, Iowa, and Connecticut. Low rates are increasing for females in New Mexico and Utah. Some of these areas are associated with higher smoking percentages (Kentucky, greater Georgia, and Detroit). Adenocarcinoma rates for males are increasing for half of the SEER areas in the current analysis and nearly all of the SEER areas among females, often tied to areas with higher current smoking. High adenocarcinoma rates for both males and females tend to be in areas where there is high smoking and areas low for no insurance (i.e., high coverage areas). Adenocarcinoma is increasing in counties with relatively high populations of blacks, American Indian/Alaska Natives, APIs, and Hispanics. An increase in adenocarcinoma for females was detected in the previous analysis (1) but the increase in trend began 1 year earlier, in 2004, and an increase was detected for males as well.

Lung cancer incidence rates by histologic type and geospatial distribution are important to consider as rates for the subtypes may have a different explanation depending on the time frame, population, and cofactors. Other exposures, including potential exposure to tobacco products or SHS are a consideration; however, the data used in the models do not include any direct measurements of exposure to tobacco products. It is important to note differences by geography as an indicator of health behaviors, including tobacco free zones, and the need for messages on prevention. This paper is one of the first to discuss lung cancer incidence rates by histologic type on a national population-based level and to model those rates using cofactors that help explain geospatial differences.

These results will further refine surveillance and control efforts for lung cancer by type. It is clear that not all subtypes of lung cancer appear in the US population to the same extent; there are differences based on subtype, geography, demographics, SES, exposure to tobacco products, and possibly other health behaviors. Although the results for smoking are at the area level and not the individual level, the results are what would be expected, and perhaps the area level results for other variables would be consistent as well. Future studies should continue to monitor rates of lung cancer by histologic subtype and assess access to treatment as well as survival.

## Ethics Approval and Consent to Participate

The cancer data used in this analysis are derived from the SEER incident cancer database. These data are collected through the participating SEER cancer registries. Consent for publication: no individual identifying data are contained in this analysis.

## Availability of Data and Materials

The data used in this analysis are available publicly through the SEER program, http://seer.cancer.gov/data/access.html. The SEER18 incidence November 2013 submission file was used for diagnosis years 2000 to 2011, excluding Alaska Natives.

## Author Contributions

DL conceived of the analysis, coordinated the project, contributed to the analysis and content, and drafted the manuscript. LP and LZ conceived the specific geospatial analysis and conducted the modeling. LP contributed the micromap plots. All authors contributed to the data interpretation, and read, edited, and approved the final manuscript.

## Conflict of Interest Statement

The authors declare that they have no competing interests. This work was entirely sponsored by the National Cancer Institute, National Institutes of Health, US Department of Health, and Human Services and is therefore in the public domain. At no time, third party payments were received. The authors have no conflicting financial interests to report.

## Footnotes

^1^*Current Cigarette Smoking Among Adults in the United States*. Available from: https://www.cdc.gov/tobacco/data_statistics/fact_sheets/adult_data/cig_smoking/

^2^*The R Project for Statistical Computing*. Available from: https://www.r-project.org/

## References

[1] Lewis DR Check D Caporaso NE Travis WD Devesa SS. US lung cancer trends by histologic type. Cancer (2014) 120:2883–92. 10.1002/cncr.28749 25113306 PMC4187244

[2] National Cancer Institute. Overview of the SEER Program. Surveillance, Epidemiology, and End Results (SEER) Program (2016). Available from: https://seer.cancer.gov/about/overview.html

[3] Hosgood H Farah C Black CC Schwenn M Hock JM. Spatial and temporal distributions of lung cancer histopathology in the state of Maine. Lung Cancer (2013) 82:55–62. 10.1016/j.lungcan.2013.06.018 23910905

[4] CDC. National Health Interview Survey, 1997-June 2015, Sample Adult Core Component (2015). Available from: https://www.cdc.gov/nchs/data/nhis/earlyrelease/earlyrelease201511_08.pdf

[5] Jemal A Homa DM O’Connor E Babb SD Caraballo RS Singh T Current cigarette smoking among adults – United States, 2005-2014. MMWR Morb Mortal Wkly Rep (2015) 64(44):1233–40. 10.15585/mmwr.mm6444a2 26562061

[6] Khuder S. Effect of cigarette smoking on major histological types of lung cancer: a meta-analysis. Lung Cancer (2001) 31:139–48. 10.1016/S0169-5002(00)00181-1 11165392

[7] Baldini EH Strauss GM. Women and lung cancer. Waiting to exhale. Chest (1997) 112:229S–34S. 10.1378/chest.112.4_Supplement.229S 9337294

[8] Ferguson MK Wang J Hoffman PC Haraf DJ Olak J Masters GA Sex-associated differences in survival of patients undergoing resection for lung cancer. Ann Thorac Surg (2000) 69:245–50. 10.1016/S0003-4975(99)01078-4 10654523

[9] Howlader N Noone AM Krapcho M Garshell J Miller D Altekruse S SEER Cancer Statistics Review, 1975-2012. Bethesda, MD: National Cancer Institute (2015).

[10] Kim HJ Fay MP Feuer EJ Midthune DN. Permutation tests for joinpoint regression with applications to cancer rates. Stat Med (2000) 19(3):335–51. 10.1002/(SICI)1097-0258(20000215)19:3<335::AID-SIM336>3.3.CO;2-Q 10649300

[11] SAS Institute Inc. SAS/STAT(R) 9.2 User’s Guide. 2nd ed Cary, NC: SAS Institute Inc. (2009).

[12] Pickle LW Pearson JB Carr DB micromapST: exploring and communicating geospatial patterns in US State Data. J Stat Softw (2015) 63(3):1–25. 10.18637/jss.v063.i03

[13] Yu M Tatalovich Z Gibson JT Cronin KA. Using a composite index of socioeconomic status to investigate health disparities while protecting the confidentiality of cancer registry data. Cancer Causes Control (2014) 25(1):81–92. 10.1007/s10552-013-0310-1 24178398

[14] Friedman J Hastie T Tibshirani R. Regularization paths for generalized linear models via coordinate descent. J Stat Softw (2010) 33(1):1–22. 10.18637/jss.v033.i01 20808728 PMC2929880

[15] U.S. Census Bureau. Comparing 2009 American Community Survey Data, 2013 (2017). Available from: https://www.census.gov/acs/www/data/data-tables-and-tools/american-factfinder/

[16] National Cancer Institute. SEER*Stat Software (2016). Available from: http://seer.cancer.gov/seerstat/

[17] Waller LA Gotway CA Applied Spatial Statistics for Public Health Data. Hoboken, NJ: John Wiley & Sons (2004).

[18] Akaike H New look at statistical-model identification. IEEE Trans Automat Contr (1974) 19(6):716–23. 10.1109/TAC.1974.1100705

[19] Bishop J Teruya-Feldstein J Westra W Pelosi G Travis W Rekhtman N p40 (DeltaNp63) is superior to p63 for the diagnosis of pulmonary squamous cell carcinoma. Mod Pathol (2012) 25:405–15. 10.1038/modpathol.2011.173 22056955

[20] Daniels M Bowman R Yang I Govindan R Fong K. An emerging place for lung cancer genomics in 2013. J Thorac Dis (2013) 5(Suppl 5):s491–7. 10.3978/j.issn.2072-1439.2013.10.06 24163742 PMC3804884

[21] Travis W Brambilla E Noguchi M Nicholson A Geisinger K Yatabe Y International Association for the Study of Lung Cancer/American Thoracic Society/European Respiratory Society international multidisciplinary classification of lung adenocarcinoma: executive summary. Proc Am Thorac Soc (2011) 8(5):381–5. 10.1513/pats.201107-042ST 21926387

[22] Travis W Brambilla E Riely G. New pathologic classification of lung cancer: relevance for clinical practice and clinical trials. J Clin Oncol (2013) 31:992–1001. 10.1200/JCO.2012.46.9270 23401443

[23] Diggle PJ Liang K-Y Zeger SL Analysis of Longitudinal Data. Oxford, UK: Oxford University Press (1994).

[24] Howlader N Noone AM Krapcho M Garshell J Neyman N Altekruse SF, editors. SEER Cancer Statistics Review, 1975-2010. Bethesda, MD: National Cancer Institute (2013). Available from: http://seer.cancer.gov/csr/1975_2010/

[25] Travis WD Brambilla E Burke AP Marx A Nicholson AG, editors. WHO Classification of Tumours of the Lung, Pleura, Thymus and Heart. Lyon, France: International Agency for Research on Cancer (2015). 10.1097/JTO.0000000000000663 26291007

[26] Thun MJ Carter BD Feskanich D Freedman ND Prentice R Lopez AD 50-year trends in smoking-related mortality in the United States. N Engl J Med (2013) 368:351–64. 10.1056/NEJMsa1211127 23343064 PMC3632080

[27] Pierce JB White MM Messer K. Changing age-specific patterns of cigarette consumption in the United States, 1992-2002: association with smoke-free homes and state-level tobacco control activity. Nicotine Tob Res (2009) 11:171–7. 10.1093/ntr/ntp014 19246423 PMC2658899

[28] Samet JM Avila-Tang E Boffetta P Hannan LM Olivo-Marston S Thun MJ Lung cancer in never smokers: clinical epidemiology and environmental risk factors. Clin Cancer Res (2009) 15:5626–45. 10.1158/1078-0432.CCR-09-0376 19755391 PMC3170525

[29] Tota JE Ramanakumar AV Franco EL. Lung cancer screening: review and performance comparison under different risk scenarios. Lung (2014) 192:55–63. 10.1007/s00408-013-9517-x 24153450

[30] Yu M Feuer EJ Cronin KA Caporaso NE. Use of multiple imputation to correct for bias in lung cancer incidence trends by histologic subtype. Cancer Epidemiol Biomarkers Prev (2014) 23:1546–58. 10.1158/1055-9965.EPI-14-0130 24855099 PMC4119525

[31] Dominici F McDermott A Zeger SL Samet JM. National maps of the effects of particulate matter on mortality: exploring geographical variation. Environ Health Perspect (2003) 111:39–43. 10.1289/ehp.5181 12515677 PMC1241304

[32] Samet JM Zeger SL Dominici F Dockery D Schwartz J The National Morbidity, Mortality, and Air Pollution Study (HEI Project No. 96-7): Methods and Methodological Issues. Cambridge, MA: Health Effects Institute (2000). 11098531

[33] Samet JM Zeger SL Dominici F Curriero F Coursac I Dockery D The National Morbidity, Mortality, and Air Pollution Study (HEI Project No. 96-7): Morbidity and Mortality from Air Pollution in the United States. Cambridge, MA: Health Effects Institute (2000). 11354823

[34] Zeger SL Dominici F McDermott A Samet JM. Mortality in the medicare population and chronic exposure to fine particulate air pollution in urban centers (2000-2005). Environ Health Perspect (2008) 116:1614–9. 10.1289/ehp.11449 19079710 PMC2599753

[35] Pope CA Burnett RT Thun MJ Calle EE Krewski D Ito K Lung cancer, cardiopulmonary mortality, and long-term exposure to fine particulate air pollution. JAMA (2002) 287:1132–41. 10.1001/jama.287.9.1132 11879110 PMC4037163

[36] Correia AW Pope CA Dockery DW Wang Y Ezzati M Dominici F. Effect of air pollution control on life expectancy in the United States. An analysis of 545 U.S. counties for the period from 2000 to 2007. Epidemiology (2013) 24(1):23–31. 10.1097/EDE.0b013e3182770237 23211349 PMC3521092

[37] Gharibvand L Shavlik D Ghamsary M Beeson WL Soret S Knutsen R The association between ambient fine particulate air pollution and lung cancer incidence: results from the AHSMOG-2 study. Environ Health Perspect (2017) 125(3):378–84. 10.1289/EHP124 27519054 PMC5332173

[38] Pickle LW Mungiole M Jones GK White AA, editors. Atlas of United States Cancer Mortality. Hyattsville, MD: National Center for Health Statistics (1996).

