# Supplementary material for: Recent Spatiotemporal Patterns of US Lung Cancer by Histologic Type
Source: Front Public Health. 2017 May 19;5:82. doi: 10.3389/fpubh.2017.00082 (PMC5437205; doi:10.3389/fpubh.2017.00082)

Supplemental Figure. Total lung cancer mortality rates by county in the US. From top row left clockwise: total lung cancer for males for 2002-2004; total lung cancer for males for 2005-2008; total lung cancer for females for 2005-2008; total lung cancer for females for 2002-2004. Mortality rates are per 100,000 population.

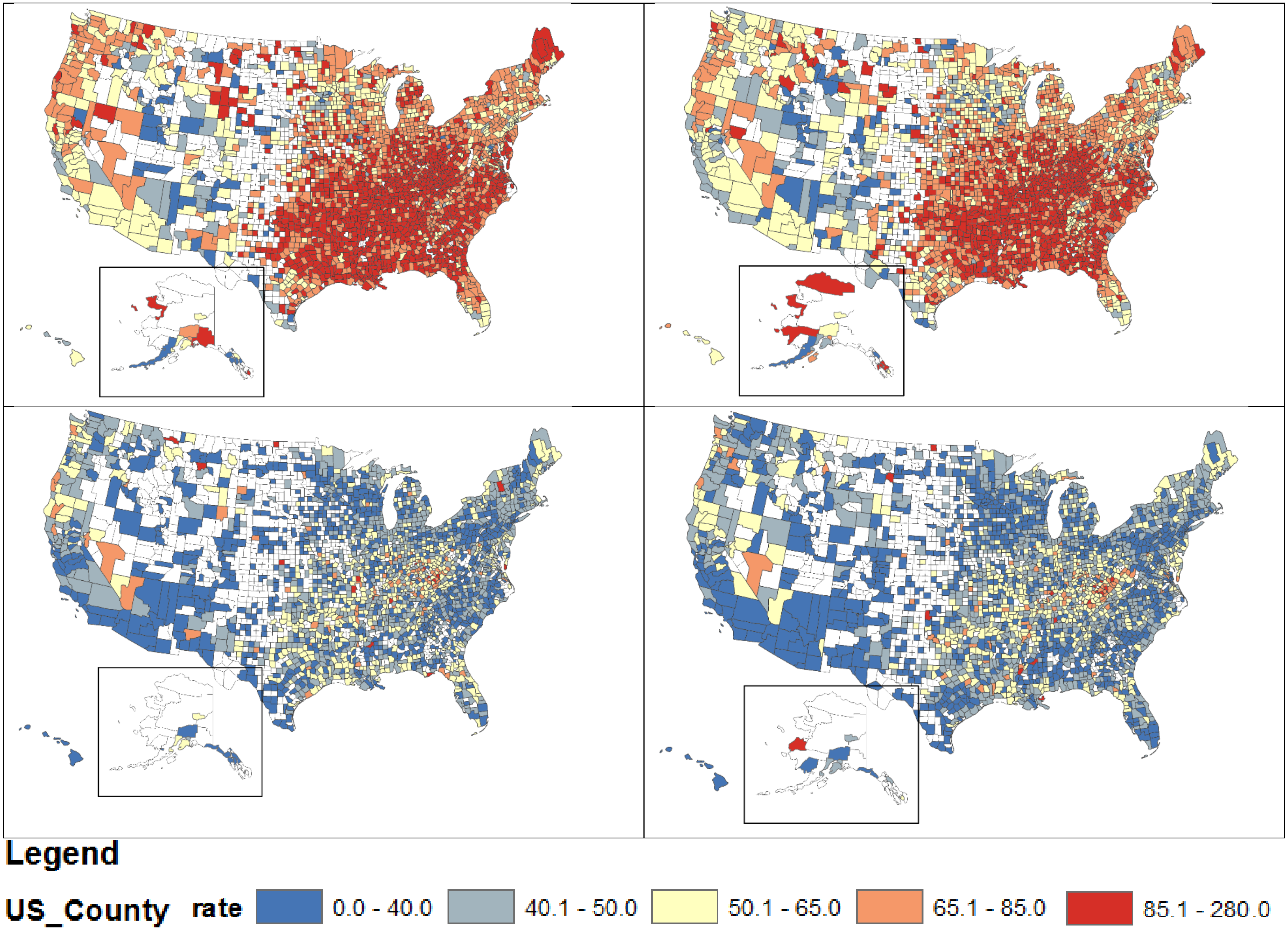

Supplement: Supplementary file 1 [file image_1.pdf]
